# Investigating the Effect of Vibration Signal Length on Bearing Fault Classification Using Wavelet Scattering Transform

**DOI:** 10.3390/s25030699

**Published:** 2025-01-24

**Authors:** Suparerk Janjarasjitt

**Affiliations:** Department of Electrical and Electronic Engineering, Ubon Ratchathani University, 85 Sathonlamak, Warin Chamrap, Ubon Ratchathani 34190, Thailand; suparerk.j@ubu.ac.th

**Keywords:** wavelet scattering transform, vibration signal, bearing fault, condition monitoring, support vector machine

## Abstract

Bearing condition monitoring and prognosis are crucial tasks for ensuring the proper operation of rotating machinery and mechanical systems. Vibration signal analysis is one of the most effective techniques for bearing condition monitoring and prognosis. In this study, the wavelet scattering transform, derived from wavelet transforms and incorporating concepts from scattering transform and convolutional network architectures, was utilized to extract quantitative features from vibration signals. The number of wavelet scattering coefficients obtained from vibration signals of different lengths varied due to the use of predefined wavelet and scaling filters in the wavelet scattering network. Additionally, these wavelet scattering coefficients are associated with different scattering paths within the corresponding wavelet scattering networks. Eight different lengths of vibration signals, associated with fifteen classes of rolling element bearing faults and conditions, were investigated using wavelet scattering feature extraction. The classes of rolling element bearing faults and conditions included normal bearings as well as ball and inner race faults with various fault diameters ranging from 0.007 inches to 0.028 inches. For the specific dataset validated, the computational results indicated that excellent bearing fault classification was achievable using wavelet scattering feature vectors derived from vibration signals with lengths of at least 6000 samples.

## 1. Introduction

Rolling element bearings operate by positioning rolling elements, such as balls or rollers, between two concentric rings known as races. These races carry the load while minimizing friction and wear. Rolling element bearings are pivotal and versatile components in rotating machinery, serving as critical intermediaries in load-bearing and facilitating smooth, low-friction movement between machine parts. Rolling element bearings are extensively utilized in a wide range of applications, including industrial machinery, renewable energy, aerospace, automotive vehicles, railroads, agricultural equipment, and construction equipment. In particular, motors are among the machinery that utilize bearings. Further, they play an important role in powering mechanical systems by converting electrical energy into mechanical motion.

The health and condition of bearings are crucial factors that affect the performance of motors and the overall operational efficiency of mechanical systems. Therefore, monitoring the health and condition of bearings is essential to prevent unexpected failures, reduce maintenance costs, and ensure the reliability and longevity of machinery. There are several techniques that can be employed for monitoring the health and condition of bearings. Key methods for bearing health and condition monitoring and prognosis include vibration signal analysis, ultrasound, acoustic emission, infrared thermography, oil composition and contamination analysis, current signature analysis, electrical discharge monitoring, and magnetic flux measurement [1,2,3]. Each of these methods offers distinct advantages and disadvantages.

Vibration signal analysis is one of the most extensively studied and widely applied methods for bearing condition monitoring and prognosis. The primary reasons that make vibration signal analysis the most common method for bearing condition monitoring and prognosis include its non-destructive nature, high sensitivity to a wide range of bearing fault types and severities, and its capability for early detection and real-time monitoring. The characteristics of vibration reflect the condition of bearings. Distinct vibration patterns provide insights into specific anomalies and defects in bearings, such as inner race faults, outer race faults, and rolling element defects. Vibration signals can be captured by sensors [4] such as piezoelectric and accelerometers.

Numerous computational tools and techniques have been developed and applied to vibration signals for bearing condition monitoring and prognosis. Originally, a signal processing technique for vibration signal analysis was derived from mathematical models of vibration signals corresponding to bearings and their constituent components, leading to the development of envelope analysis and spectral analysis of vibration signals [5]. In addition, various computational tools and techniques derived from time-domain analysis and frequency-domain analysis were applied to extract quantitative features of vibration signals and used for bearing condition monitoring and prognosis. However, the primary drawback of these computational tools and techniques is their difficulty in handling the non-stationary characteristics of vibration signals.

In general, two approaches are commonly employed in novel and advanced computational methods for bearing condition monitoring and prognosis. The first is a conventional approach, consisting of two main stages: feature extraction and classification. Computational tools and techniques are used to extract quantitative features from vibration signals, which are then classified to identify the corresponding bearing condition. Recently, most classifiers applied have been derived from machine learning techniques. Another approach involves utilizing modern computational tools and techniques based on deep learning algorithms. These tools and techniques can be directly applied to raw vibration signals or processed vibration signals.

In addition to Fourier transforms and spectral analysis, common computational tools and techniques have been applied to process vibration signals and extract distinct features for bearing condition monitoring and bearing fault classification include time-frequency analysis [6,7], wavelet transforms [8,9,10,11] and their variants such as wavelet packet transform [12], empirical mode decomposition (EMD) [13], variation mode decomposition (VMD) [14], hidden Markov models [15,16], and computational algorithms derived from nonlinear dynamical analysis and chaos theory [1,17,18]. Support vector machine (SVM) is one of the most commonly used machine learning algorithms [2,3] for identifying bearing conditions and classifying bearing faults using various features extracted from vibration signals [17,18,19,20]. Other machine learning algorithms, such as random forest [21,22] and k-nearest neighbor [23,24,25], are also employed. In many recent studies, a variety of deep learning algorithms and neural network architectures, including convolutional neural network (CNN) [26,27], recurrent neural network (RNN) [28], and autoencoder [29], have been developed and applied to vibration signals for bearing condition monitoring and bearing fault classification.

Despite the application of various computational tools and techniques derived from various theories and concepts to vibration signals for feature extraction and bearing fault classification, ongoing efforts aim to develop novel computational tools and techniques to enhance the characterization of vibration signals and improve bearing fault classification. In this study, the wavelet scattering transform, derived upon wavelet transforms with a combination of the concept of scattering transform and convolutional network architectures [30,31,32,33], was utilized to extract distinctive features from vibration signals. The vibration signals analyzed were sourced from the CWRU Bearing Dataset, a widely recognized benchmark extensively used for validating methods in bearing condition monitoring and fault classification [14]. The two primary research questions addressed in this study were: (1) how the length of vibration signals influenced the wavelet scattering transform, and (2) how the corresponding wavelet scattering features affected the performance of bearing fault classification. As the wavelet scattering network generally employs predefined wavelet and scaling filters, the application of these wavelet scattering networks to vibration signals varied according to the length of vibration signals. The performance of bearing fault classifications using wavelet scattering features extracted from various lengths of vibration signals was examined and validated using a support vector machine (SVM) as a multi-class classifier to identify the corresponding bearing condition and fault. Accordingly, the computational results obtained in this study reveal the optimal length of vibration signals required for achieving accurate bearing fault classification.

## 2. Materials and Methods

### 2.1. CWRU Bearing Dataset

A dataset provided by the CWRU Bearing Data Center, available at https://engineering.case.edu/bearingdatacenter (accessed on 12 November 2024), contains ball bearing test data for various bearing defects and conditions as well as normal bearings. The vibration data were collected using accelerometers which were attached to the housing with magnetic bases and acquired using a 16-channel DAT recorder. In particular, for the vibration data examined in this study, the accelerometers were placed at the 12 o’clock position at the drive end of the motor housing. A test bearing system consists of a 2-HP Reliance Electric motor, a torque transducer/encoder, a dynamometer, and a control circuit.

Faulty bearings were located at both the drive end and the fan end of the test bearing system. Single point defects were separately introduced into the test motor bearings using electro-discharge machining (EDM). There were three main types of ball bearing defects: ball faults, inner race faults, and outer race faults (located at 3 o’clock, 6 o’clock, and 12 o’clock). The fault diameters include 0.007 inches (7 mils), 0.014 inches (14 mils), 0.021 inches (21 mils), and 0.028 inches (28 mils). In the bearing experiments, motor loads were set to 0, 1, 2, and 3 HP, while motor speeds ranged from 1720 to 1797 RPM. The vibration data associated with ball bearing defects, including ball faults, inner race faults, and outer race faults, were digitized and recorded at a sampling frequency of 12,000 Hz. In contrast, the vibration data of normal (healthy) bearings were digitized and recorded at a sampling frequency of 48,000 Hz.

### 2.2. Vibration Signal Segmentation

In this study, vibration data specifically acquired from the accelerometers located at the drive end and associated with fifteen bearing conditions including normal, ball faults at the drive end with fault diameters of 0.007, 0.014, 0.021 and 0.028 inches, ball faults at the fan end with fault diameters of 0.007, 0.014 and 0.021 inches, inner race faults at the drive end with fault diameters of 0.007, 0.014, 0.021 and 0.028 inches, and inner race faults at the fan end with fault diameters of 0.007, 0.014 and 0.021 inches were examined. Class labels were assigned for each bearing condition as follows: normal (NORM), ball faults at the drive end with fault diameters of 0.007, 0.014, 0.021, and 0.028 inches (BA07D, BA14D, BA21D, and BA28D), ball faults at the fan end with fault diameters of 0.007, 0.014, and 0.021 inches (BA07F, BA14F, and BA21F), inner race faults at the drive end with fault diameters of 0.007, 0.014, 0.021, and 0.028 inches (IR07D, IR14D, IR21D, and IR28D), and inner race faults at the fan end with fault diameters of 0.007, 0.014, and 0.021 inches (IR07F, IR14F, and IR21F). All fifteen bearing conditions and their corresponding class labels used in this study are summarized in Table 1.

The vibration signals of normal bearings were decimated to reduce the sampling frequency from 48,000 Hz to 12,000 Hz, matching the sampling frequency of the vibration signals associated with bearing and inner race faults. For the decimation, the 5th-order Chebyshev Type I lowpass filter was applied as an anti-aliasing filter prior to downsampling by a factor of four. A vibration signal associated with each bearing condition was segmented into vibration signal epochs with eight different epoch lengths: 600, 900, 1200, 3000, 6000, 9000, 12,000, and 15,000 samples, respectively, corresponding to 0.05, 0.075, 0.10, 0.25, 0.5, 0.75, 1.00, and 1.25 s. The sliding step for vibration signal segmentation is set to half the epoch length, resulting in a 50% overlap. Accordingly, the numbers of vibration signal epochs corresponding to various epoch lengths are summarized in Table 2. The number of vibration signal epochs from normal bearings ranges between 1410 and 52 while the number of vibration signal epochs from faulty bearings ranges between 1622 and 60. The total numbers of vibration signal epochs with length of 600, 900, 1200, 3000, 6000, 9000, 12,000, and 15,000 samples are 24,021, 15,985, 11,965, 4730, 2320, 1517, 1130, and 892, respectively.

**Figure 1:**
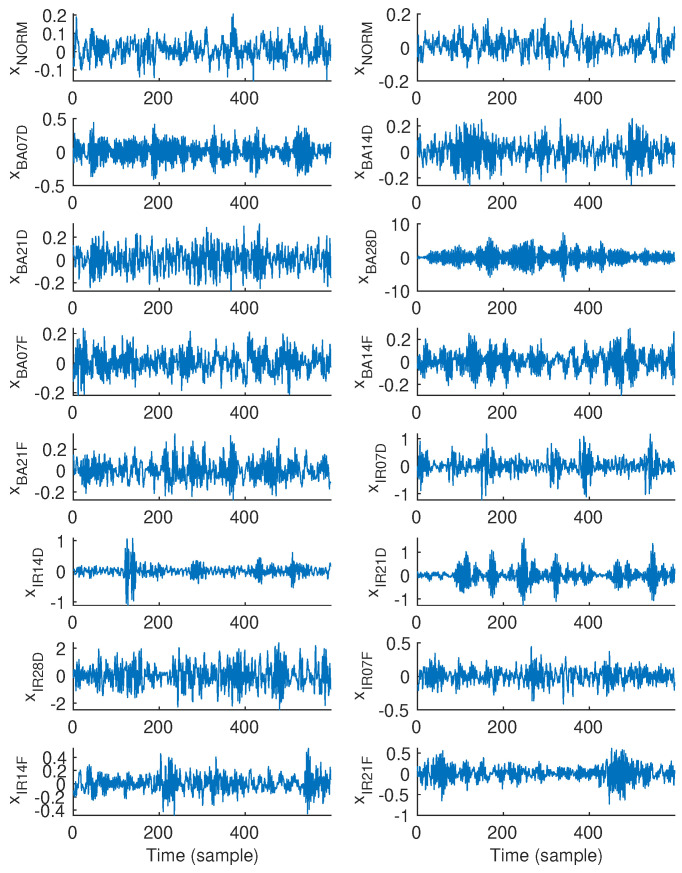
Exemplary vibration signal epochs, each with a length of 600 samples, associated with each class.

**Figure 2:**
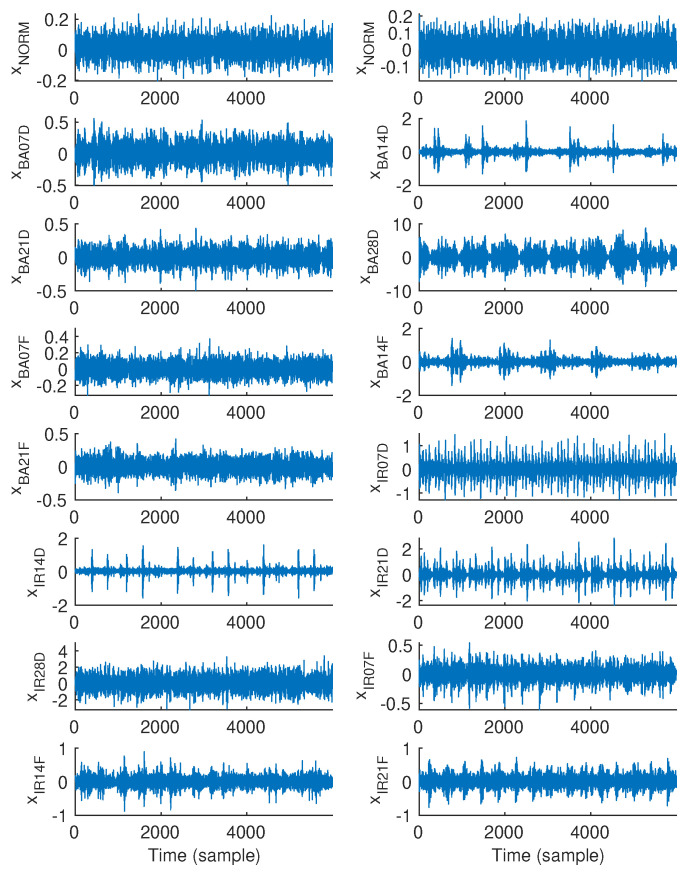
Exemplary vibration signal epochs, each with a length of 6000 samples, associated with each class.

**Figure 3:**
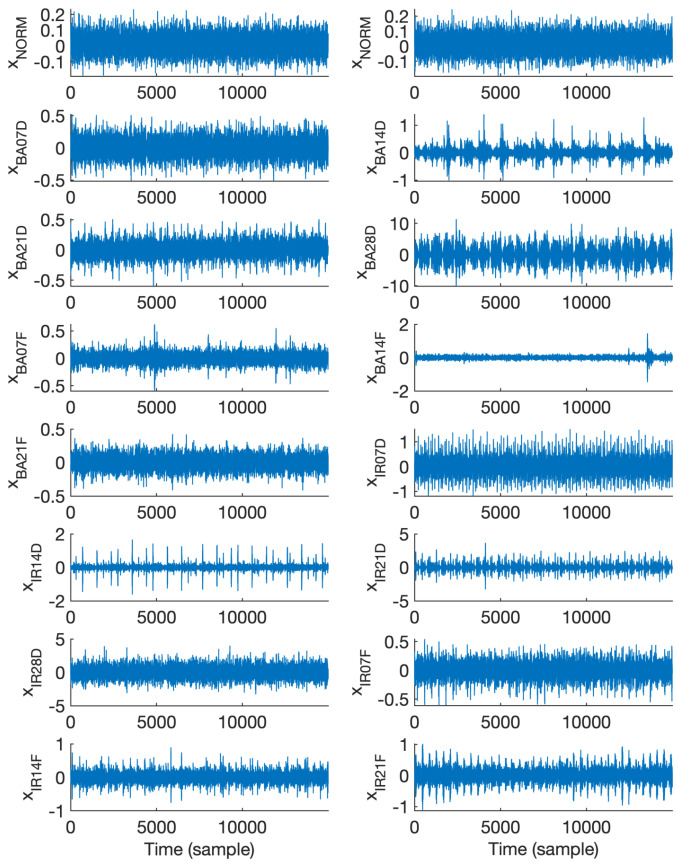
Exemplary vibration signal epochs, each with a length of 15,000 samples, associated with each class.

Exemplary 600-sample vibration signal epochs associated with all fifteen classes, i.e., NORM, BA07D, BA14D, BA21D, BA28D, BA07F, BA14F, BA21F, IR07D, IR14D, IR21D, IR28D, IR07F, IR14F, and IR21F, which were randomly chosen are illustrated in Figure 1. Similarly, Figure 2 and Figure 3 show exemplary vibration signal epochs associated with all fifteen classes with the length of 6000 and 15,000 samples, respectively.

### 2.3. Wavelet Scattering Feature Extraction

Wavelet scattering transform is a computational framework that leverages the wavelet transform and deep convolutional network architectures [30,31,32,33]. The wavelet transform of *x* computes a convolution of *x* with a lowpass filter ϕ and convolutions with all higher-frequency wavelets $\psi_{\lambda }$, corresponding to scale $2^{J}$ [30,31]

(1) $$W\left\{x\right\}={\left\{x*\phi \left(u\right),x*\psi_{\lambda }\left(u\right)\right\}}_{\lambda \in \Lambda }$$

where $\psi_{\lambda }\left(u\right)=2^{-2j}\psi \left(2^{-j}r^{-1}u\right)$ with $\lambda =2^{-j}r$ and the convolution is defined by $x*\psi_{\lambda }\left(u\right)=\int x\left(u\right)\psi_{\lambda }\left(t-u\right)du$. Wavelets are stable to deformations as they are localized waveforms [32]. The convolution is however not translation invariant. The wavelets $\psi_{\lambda }$ and the lowpass filter ϕ typically span the whole frequency space. A wavelet scattering network cascades wavelet transform convolutions with nonlinear modulus and averaging operators [32]. A scattering transform along the path *p* is defined as an integral, normalized by the response of a Dirac [32]

(2) $$\bar{S}x\left(p\right)=\mu_{p}^{-1}\int U\left[p\right]x\left(u\right)du\mathrm{with}\mu_{p}=\int U\left[p\right]\delta \left(u\right)du$$

where $\bar{S}x\left(p\right)$ denotes the scattering coefficients. A windowed scattering transform in the neighborhood of *u* is defined by [32]

(3) $$S\left[p\right]x\left(u\right)=U\left[p\right]x*\phi_{2^{J}}\left(u\right)=\int U\left[p\right]x\left(v\right)\phi_{2^{J}}\left(u-v\right)dv$$

and, accordingly,

(4) $$S\left[p\right]x\left(u\right)=||\cdots |x*\psi_{\lambda_{1}}|*\psi_{\lambda_{2}}\left|\cdots *\psi_{\lambda_{m}}\right|*\phi_{2^{J}}\left(u\right)$$

with $S\left[0\right]x=x*\phi_{2^{J}}$. The windowed scattering coefficient of order *m*, S[p]x(u), is computed at the layer *m* of a convolutional network where $p=\left(\lambda_{1},\lambda_{2},\ldots ,\lambda_{m}\right)$ is the path of length *m*. As opposed to conventional convolutional networks, output scattering coefficients are generated by each layer rather than the last layer [34]. For proper wavelets, the first network layer yields SIFT-type descriptor, i.e., the first-order coefficients $S[\lambda_{1}]x$ are equivalent to SIFT coefficients, while the next network layers provide complementary invariant information [32].

The wavelet scattering transform is applied to vibration signal epochs using the analytic Morlet wavelet. A corresponding network for wavelet scattering is composed of two filter banks where the first filter bank has a quality factor of eight wavelets per octave and the second filter bank has a quality factor of two wavelets per octave. In addition, an invariance scale of the scattering transform is equal to a half of the epoch length, i.e., 300, 450, 600, 1500, 3000, 4500, 6000, and 7500 sample for the vibration signal epochs with the length of 600, 900, 1200, 3000, 6000, 9000, 12,000, and 15,000 samples, respectively.

For vibration signal epochs with lengths of 600, 900, 1200, 3000, 6000, 9000, 12,000, and 15,000 samples, the corresponding numbers of scattering paths are 65, 97, 101, 179, 255, 303, 347, and 401, respectively. Additionally, the numbers of scattering coefficients obtained from each scattering path are 10, 8, 10, 12, 12, 9, 12, and 8 for the respective epoch lengths. Consequently, the total numbers of wavelet scattering coefficients obtained from vibration signal epochs with lengths of 600, 900, 1200, 3000, 6000, 9000, 12,000, and 15,000 samples are, respectively, 650, 776, 1010, 2148, 3060, 2727, 4164, and 3208. Table 3 summarizes the description of corresponding wavelet scattering networks and the number of wavelet scattering features.

In addition to bearing fault classifications, the minimum redundancy maximum relevance (MRMR) algorithm was utilized to explore the predominance of wavelet scattering features. The MRMR algorithm is a feature selection technique that ranks key features or provides an optimal set of features by effectively minimizing the redundancy of a feature set and maximizing its relevance to the response variable. The MRMR algorithm quantifies the redundancy and relevance using the mutual information where the mutual information *I* of two variables, *x* and *y*, is defined based on the joint probabilistic distribution p(x,y) and the respective marginal probabilities p(x) and p(y) [35]:

(5) $$I\left(x,y\right)=\sum_{i,j}p\left(x_{i},y_{j}\right)\log\left(\frac{p(x_{i},y_{j})}{p\left(x_{i}\right)p\left(y_{j}\right)}\right).$$

The MRMR feature set [35] is obtained by optimizing both the minimum redundancy condition:

(6) $$\min\;\frac{1}{{\left|S\right|}^{2}}\sum_{u_{i},v_{j}\in S}I\left(u_{i},v_{j}\right)$$

where |S| denotes the number of features in the subset of features *S* and the maximum relevance condition:

(7) $$\max\;\frac{1}{\left|S\right|}\sum_{i\in S}I\left(h,u_{i}\right)$$

where *h* denotes the classification variable or targeted classes, i.e., $h=\{h_{1},h_{2},\ldots ,h_{K}\}$.

### 2.4. Bearing Fault Classification and Performance Evaluation

Feature vectors consisting of all wavelet scattering coefficients of the vibration signal epochs, without any dimensionality reduction, were applied for bearing fault classification. A size of the wavelet scattering feature vectors thus varied according to the length of the vibration signal epochs. A support vector machine (SVM) used as a multi-class classifier was applied for classifying the corresponding class of wavelet scattering feature vectors of the vibration signal epochs. The SVM is a supervised learning algorithm [36,37,38] commonly used for classification and regression applications. The primary goal of the SVM is to find an optimal hyperplane that separates data points in different classes with the maximum margin [39]. This hyperplane is determined by the support vectors, which are the data points closest to the decision boundary. For a given training set $S=\{\left(x_{1},y_{1}\right),...,\left(x_{l},y_{l}\right)\}$ of data points $x_{i}$ with corresponding labels $y_{i}$, the SVM finds a hyperplane that is defined by [39]

(8) $$\langle w,\phi \left(x_{i}\right)\rangle =b$$

such that the margin

(9) $$\gamma =\min_{1\leq i\leq l}y_{i}\left(\langle w,\phi \left(x_{i}\right)\rangle -b\right)$$

is maximized where 〈·,·〉 denotes the inner product, w is a *l*-dimensional weight vector, and *b* is a bias.

For linearly separable classes, there exists a hyperplane (w,b) such that [39]

(10) $$y_{i}\left(\langle w,\phi \left(x_{i}\right)\rangle -b\right)\geq \gamma ,\;\forall i=1,\ldots ,l.$$

The choice of the hyperplane that maximizes the margin, by imposing ${∥w∥}^{2}=1$, is equivalent to the following optimization problem

(11) $$\max_{w,b,\gamma }\gamma$$

$$\mathrm{subject}\mathrm{to}\;y_{i}\left(\langle w,\phi \left(x_{i}\right)\rangle -b\right)\geq \gamma ,\;\forall i=1,\ldots ,l\;\mathrm{and}\;{∥w∥}^{2}=1.$$

An efficient solution can be solved in the dual space by introducing the Lagrange multipliers $\alpha_{i}$. The optimization problem in Equation (11) can then be expressed in the following dual form [39]

(12) $$\max_{\alpha }\sum_{i=1}^{l}\alpha_{i}-\frac{1}{2}\sum_{i=1}^{l}\sum_{j=1}^{l}\alpha_{i}\alpha_{j}y_{i}y_{j}\langle \phi \left(x_{i}\right),\phi \left(x_{j}\right)\rangle$$

$$\mathrm{subject}\mathrm{to}\;\sum_{i=1}^{l}\alpha_{i}y_{i}=0\;\mathrm{and}\;\alpha_{i}\geq 0.$$

Accordingly, the decision function can be expressed as

(13) $$g\left(x\right)=\mathrm{sign}\left(\sum_{i=1}^{l}\alpha_{i}y_{i}\langle \phi \left(x_{i}\right),\phi \left(x\right)\rangle -b\right).$$

The SVM offers several key advantages, including its effective handling of high-dimensional data, its capability to address nonlinear classification problems through the use of kernel functions, and its robustness against overfitting facilitated by the regularization parameter [37,38,39,40].

Two validation techniques were used to assess the performance of bearing fault classification using the wavelet scattering features of vibration signal epochs and the SVM classifier. In the first validation of bearing fault classifications, the wavelet scattering feature vectors derived from all vibration signal epochs were split into training and test subsets. The ratio of feature vectors in the training subset to the total number of feature vectors varied from approximately 3% to 96%. At each increment of the ratio of feature vectors in the training subset to the total number of feature vectors, an approximately identical portion of feature vectors from all fifteen classes were added to the training subset. The feature vectors of the training subset were used to train an SVM-based multi-class classifier, while the remaining feature vectors, corresponding to the test subset, were used to assess the performance of bearing fault classification. The overall accuracy of the bearing fault classifications obtained at each ratio of feature vectors in the training subset to the total number of feature vectors was computed. Subsequently, the learning curve in terms of classification error was determined for all ratios. For the first validation technique, a trial of computational experiments was randomized and repeated 20 times.

The second validation method used to assess the performance of bearing fault classifications was 10-fold cross validation. A set of wavelet scattering feature vectors from all classes was partitioned into ten nearly equal-sized subsets, or folds. Consequently, the bearing fault classification was divided into ten sub-classifications. For each sub-classification, a different fold of the wavelet scattering feature vectors was held out for validation, while the remaining nine folds were used for training an SVM-based multi-class classifier. The accuracy obtained from all ten sub-classifications was determined. Furthermore, the precision and the recall obtained for each class were also computed. For the second validation technique, a trial of computational experiments was randomized and repeated 40 times.

The overall accuracy of bearing fault classifications, ${\mathrm{ACC}}_{\mathrm{overall}}$, was given by

(14) $${\mathrm{ACC}}_{\mathrm{overall}}=100\times \frac{\sum_{c}\left({\mathrm{TP}}_{c}+{\mathrm{TN}}_{c}\right)}{\sum_{c}\left({\mathrm{TP}}_{c}+{\mathrm{TN}}_{c}+{\mathrm{FP}}_{c}+{\mathrm{FN}}_{c}\right)}$$

where ${\mathrm{TP}}_{c}$, ${\mathrm{TN}}_{c}$, ${\mathrm{FP}}_{c}$, and ${\mathrm{FN}}_{c}$, respectively, denote the number of true positives, the number of true negatives, the number of false positives, and the number of false negatives of a corresponding class *c*, i.e., NORM, BA07D, BA14D, BA21D, BA28D, BA07F, BA14F, BA21F, IR07D, IR14D, IR21D, IR28D, IR07F, IR14F, and IR21F. The accuracy, precision, and recall corresponding to a class *c*, ${\mathrm{Ac}}_{c}$, ${\mathrm{Pr}}_{c}$, and Rec were, respectively, given by

(15) $${\mathrm{Ac}}_{c}=100\times \frac{{\mathrm{TP}}_{c}+{\mathrm{TN}}_{c}}{{\mathrm{TP}}_{c}+{\mathrm{TN}}_{c}+{\mathrm{FP}}_{c}+{\mathrm{FN}}_{c}},$$

(16) $${\mathrm{Pr}}_{c}=100\times \frac{{\mathrm{TP}}_{c}}{{\mathrm{TP}}_{c}+{\mathrm{FP}}_{c}},\;\mathrm{and}$$

(17) Rec=100×TPcTPc+FNc.

For all bearing fault classifications and validations, the computational experiments were performed using MATLAB R2023b on a MacBook Pro (Apple Inc., Cupertino, CA, USA) with an Apple M3 Pro chip, featuring a 12-core CPU, 18-core GPU, 16-core Neural Engine, and 36 GB of memory. The second-order polynomial kernel function $K(x_{i},x_{j})$ which is given by

(18) $$K\left(x_{i},x_{j}\right)={\left(1+x_{i}^{T}x_{j}\right)}^{2},$$

with an automatic kernel scale was used for SVM models. The standardization was also applied to the wavelet scattering feature vectors of vibration signal epochs. The parameters of SVM models applied for bearing fault classifications are summarized in Table 4.

## 3. Results

### 3.1. Characteristics of Wavelet Scattering Features

The minimum redundancy maximum relevance (MRMR) algorithm was applied to rank all wavelet scattering features, i.e., wavelet scattering coefficients of vibration signal epochs, associated with various bearing classes. The twenty four top-ranked wavelet scattering features, identified using the MRMR algorithm, for vibration signal epochs with length of 600, 900, 1200, 3000, 6000, 9000, 12,000, and 15,000 samples are summarized in Table 5. A wavelet scattering feature $WS_{p,c}$ denotes the *c*th wavelet scattering coefficient obtained from the *p*th path in the corresponding wavelet scattering network. The top-ranked wavelet scattering features, identified as the most distinct by the MRMR algorithm, are unique across different epoch lengths.

**Figure 4:**
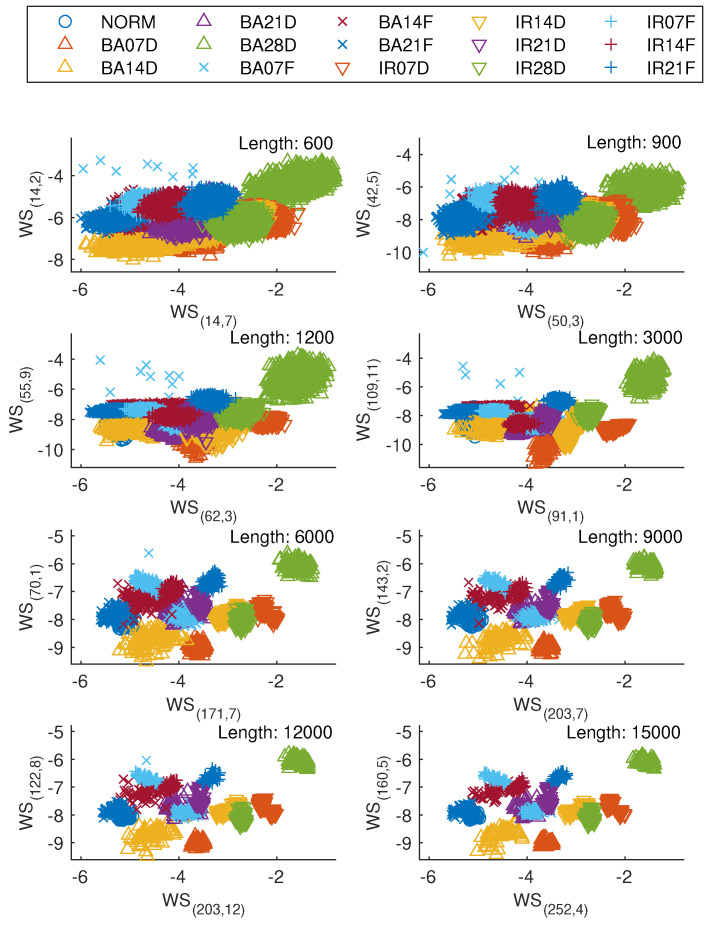
Comparison of top two wavelet scattering features of vibration signal epochs for each length.

Scatter plots shown in Figure 4 visualize the distribution of the first two wavelet scattering features of vibration signal epochs from various bearing classes corresponding to the epoch length of 600, 900, 1200, 3000, 6000, 9000, 12,000, and 15,000 sample, i.e., $WS_{\left(14,7\right)}$ and $WS_{\left(14,2\right)}$, $WS_{\left(50,3\right)}$ and $WS_{\left(42,5\right)}$, $WS_{\left(62,3\right)}$ and $WS_{\left(55,9\right)}$, $WS_{\left(91,1\right)}$ and $WS_{\left(109,11\right)}$, $WS_{\left(171,7\right)}$ and $WS_{\left(70,1\right)}$, $WS_{\left(203,7\right)}$ and $WS_{\left(143,2\right)}$, $WS_{\left(203,12\right)}$ and $WS_{\left(122,8\right)}$, and $WS_{\left(252,4\right)}$ and $WS_{(160,5}$, respectively. It was demonstrated that the wavelet scattering features from different bearing classes exhibit a tendency to be separable. Moreover, an increase in the length of vibration signal epochs enhances the differentiability of the corresponding wavelet scattering features.

### 3.2. Performance of the Bearing Fault Classifications

**Figure 5:**
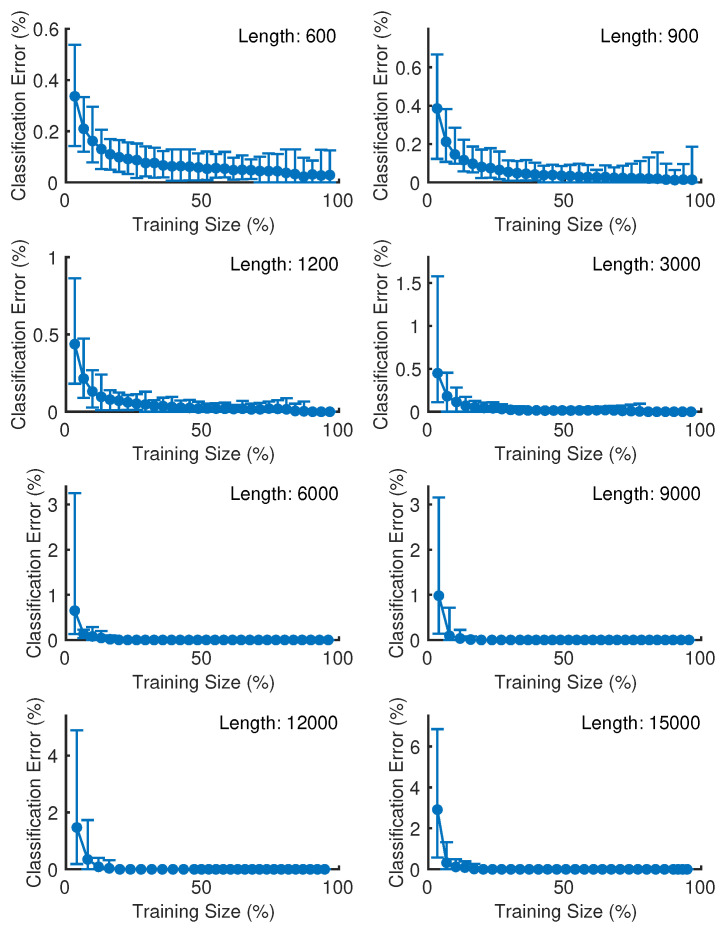
Learning curve of the bearing fault classifications for each epoch length.

From the first validation, i.e., training-test subset split, the average values of classification errors and the corresponding maximum and minimum values obtained from the bearing fault classifications using the wavelet scattering feature vectors of vibration signal epochs with the length of 600, 900, 1200, 3000, 6000, 9000, 12,000, and 15,000 samples were shown in Figure 5. The circle marker specifies the mean bearing fault classification error of all fifteen bearing classes corresponding to each training size, i.e., a ratio of wavelet scattering feature vectors in the training subset to the total number of wavelet scattering feature vectors. The top and bottom bars indicate the corresponding maximum and minimum bearing fault classification error, respectively. The bearing fault classification errors tend to decrease as the training size increases. However, overfitting characteristics were observed when the length of vibration signal epochs was too small (600, 900, 1200, and 3000 samples).

The error of the bearing fault classifications can be zero for all repeated trials when the length of vibration signal epochs was greater than 900 samples, i.e., 1200, 3000, 6000, 9000, 12,000, and 15,000 samples. Furthermore, the bearing fault classification error of the bearing fault classifications using the wavelet scattering feature vectors of vibration signal epochs with lengths of 1200, 3000, 6000, 9000, 12,000, and 15,000 samples reached absolute zero when the training size was equal to 90.34%, 80.59%, 22.63%, 19.44%, 19.73%, and 20.18% of the total number of wavelet scattering feature vectors, respectively.

From the 10-fold cross validation, the average and standard deviation values of the accuracy for classifying individual classes, i.e., NORM, BA07D, BA14D, BA21D, BA28D, BA07F, BA14F, BA21F, IR07D, IR14D, IR21D, IR28D, IR07F, IR14F, and IR21F classes, using wavelet scattering feature vectors of vibration signal epochs with lengths of 900, 1200, 3000, 6000, 9000, 12,000, and 15,000 samples were summarized in Table 6. The accuracy of bearing fault classifications for the BA07D, BA28D, IR14D, IR21D, and IR21F classes was consistently zero across all repeated trials when using wavelet scattering feature vectors of vibration signal epochs of any length, i.e., 600, 900, 1200, 3000, 6000, 9000, 12,000, and 15,000 samples.

Similarly, the average and standard deviation values of the precision and the recall for these classifications of individual bearing classes were, respectively, summarized in Table 7 and Table 8. The precision of bearing fault classifications for the BA07D, BA28D, IR14D, IR21D, and IR21F classes was consistently zero across all repeated trials when using wavelet scattering feature vectors of vibration signal epochs of any length, i.e., 600, 900, 1200, 3000, 6000, 9000, 12,000, and 15,000 samples, while the recall of bearing fault classifications was consistently zero across all repeated trials for most of the bearing classes except only the BA14D, BA21D, BA07F, and BA21F classes.

**Figure 6:**
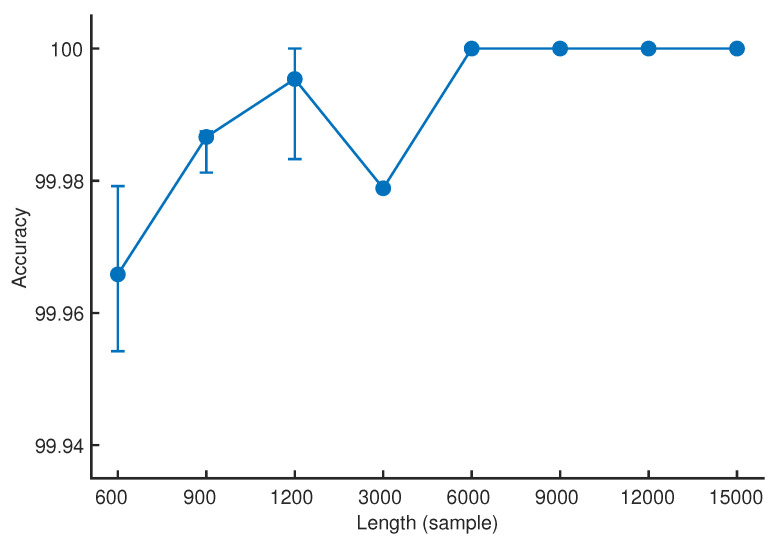
The range of overall accuracy of the bearing fault classifications for each epoch length.

The average values of overall accuracy and the corresponding maximum and minimum values obtained from the bearing fault classifications using the wavelet scattering feature vectors of vibration signal epochs with the length of 600, 900, 1200, 3000, 6000, 9000, 12,000, and 15,000 samples, indicated by the circle marker and the top and bottom bars, respectively, were depicted in Figure 6. The overall accuracy of the bearing fault classifications using the wavelet scattering feature vectors generally increased as the length of the vibration signal epochs grew, except at the epoch length of 3000 samples.

The average values of overall accuracy of bearing fault classifications using wavelet scattering feature vectors are 99.9658, 99.9866, 99.9954, 99.9789, 100.0, 100.0, 100.0, and 100.0, respectively, for the vibration signal epoch lengths of 600, 900, 1200, 3000, 6000, 9000, 12,000, and 15,000 samples. In addition, the overall accuracy of the bearing fault classifications was consistently zero across all repeated trials when using the wavelet scattering feature vectors of vibration signal epochs with the length of 6000, 9000, 12,000, and 15,000 samples. The performance of bearing fault classifications obtained using wavelet scattering feature vectors of vibration signal epochs is comparable to that reported in the literature. Table 9 summarizes the performance of bearing fault classifications using various computational methods proposed and presented in recent studies that analyzed the same dataset, namely, the CWRU Bearing Dataset. The number of bearing faults and conditions examined in those studies ranges from five to fourteen classes while the settings of computational experiments and the validation approaches varied.

## 4. Conclusions and Discussion

The performance of bearing fault classification was evaluated using quantitative features extracted from vibration signals via the wavelet scattering transform. The wavelet scattering coefficients, derived from the wavelet scattering transform of vibration signals, served as quantitative features for bearing fault classification. The dimensions of the wavelet scattering feature vectors varied with the length of vibration signal epochs, influenced by the predefined wavelet and scaling filters in the wavelet scattering network. A total of fifteen classes were applied for bearing fault classifications, corresponding to the bearing faults, fault diameters, and fault locations. From the first validation technique, the training and test subset split validation, the computational results indicated that a minimum of 20% of the total dataset was required to train SVM multi-class classifiers for achieving perfect classification across all fifteen bearing classes, when using vibration signal epochs of lengths 9000 and 12,000 samples. From the 10-fold cross-validation, perfect individual bearing fault classification for all fifteen bearing conditions was achieved based on three common performance metrics: accuracy, precision, and recall, when the length of vibration signals was greater than or equal to 6000 samples. The computational results indicated that all fifteen bearing conditions and faults were perfectly identified using 3060 wavelet scattering features extracted from vibration signals, with an optimal length of 6000 samples. This therefore suggests that defects in rolling-element bearings, including ball or inner race faults, with a minimum fault diameter of 0.007 inches can be detected within 0.50 s. It is noteworthy that the wavelet scattering transform was not significantly impacted by the decimation applied to the vibration signals of normal bearings. The wavelet scattering coefficients obtained from the original vibration signals and those obtained from the decimated vibration signals were not statistically significantly different. Furthermore, the performance of bearing fault classifications using the original vibration signals and the decimated vibration signals was substantially the same. Additionally, the performance of bearing fault classifications was not affected by various motor loads and motor speeds.

## Figures and Tables

**Table 1:** Conditions and faults of bearings.

Bearing Fault	Fault Location	Fault Diameter (inch)	Class
Normal	–	0	NORM
Ball Fault	Drive End	0.007	BA07D
		0.014	BA14D
		0.021	BA21D
		0.028	BA28D
	Fan End	0.007	BA07F
		0.014	BA14F
		0.021	BA21F
Inner Race Fault	Drive End	0.007	IR07D
		0.014	IR14D
		0.021	IR21D
		0.028	IR28D
	Fan End	0.007	IR07F
		0.014	IR14F
		0.021	IR21F

**Table 2:** Numbers of vibration signal epochs for each length.

Class	Epoch Length (Sample)							
	600	900	1200	3000	6000	9000	12,000	15,000
NORM	1410	937	702	276	136	87	66	52
BA07D	1618	1077	806	319	156	103	76	60
BA14D	1622	1079	808	320	156	104	76	60
BA21D	1621	1079	807	320	156	104	76	60
BA28D	1608	1069	801	316	156	100	76	60
BA07F	1609	1072	801	317	156	101	76	60
BA14F	1617	1077	805	319	156	103	76	60
BA21F	1611	1072	803	317	156	101	76	60
IR07D	1622	1080	808	319	156	103	76	60
IR14D	1619	1076	807	320	156	104	76	60
IR21D	1620	1078	807	320	156	104	76	60
IR28D	1611	1072	803	317	156	101	76	60
IR07F	1614	1074	804	318	156	102	76	60
IR14F	1611	1072	803	316	156	100	76	60
IR21F	1608	1071	800	316	156	100	76	60
Total	24,021	15,985	11,965	4730	2320	1517	1130	892

**Table 3:** Number of wavelet scattering coefficients for each epoch length.

Length of Vibration Signal Epochs	Number of Scattering Paths	Number of Scattering Coefficients	Number of Wavelet Scattering Coefficients
600	65	10	650
900	97	8	776
1200	101	10	1010
3000	179	12	2148
6000	255	12	3060
9000	303	9	2727
12,000	347	12	4164
15,000	401	8	3208

**Table 4:** Settings of parameters of SVM applied for bearing fault classifications.

Parameter	Attribute
Kernel function	Polynomial kernel function
Polynomial order	2
Kernel scale	Automatically determine
Box constraint	1
Standardization	True
Solver	Sequential minimal optimization (SMO) algorithm
Nu	0.5
Coding design	one-versus-one

**Table 5:** Top ranks of the wavelet scattering features for each epoch length.

Ranking	Epoch Length (Sample)							
	600	900	1200	3000	6000	9000	12,000	15,000
1	$WS_{14,7}$	$WS_{50,3}$	$WS_{62,3}$	$WS_{91,1}$	$WS_{171,7}$	$WS_{203,7}$	$WS_{203,12}$	$WS_{252,4}$
2	$WS_{14,2}$	$WS_{42,5}$	$WS_{55,9}$	$WS_{109,11}$	$WS_{70,1}$	$WS_{143,2}$	$WS_{122,8}$	$WS_{160,5}$
3	$WS_{10,7}$	$WS_{4,8}$	$WS_{14,7}$	$WS_{31,3}$	$WS_{122,9}$	$WS_{158,5}$	$WS_{193,4}$	$WS_{115,2}$
4	$WS_{7,6}$	$WS_{65,7}$	$WS_{21,7}$	$WS_{149,12}$	$WS_{21,5}$	$WS_{201,8}$	$WS_{144,6}$	$WS_{235,3}$
5	$WS_{55,10}$	$WS_{85,8}$	$WS_{14,4}$	$WS_{73,9}$	$WS_{170,9}$	$WS_{31,9}$	$WS_{114,3}$	$WS_{295,7}$
6	$WS_{43,5}$	$WS_{29,8}$	$WS_{88,7}$	$WS_{90,1}$	$WS_{237,8}$	$WS_{33,4}$	$WS_{170,10}$	$WS_{290,5}$
7	$WS_{42,1}$	$WS_{65,3}$	$WS_{81,9}$	$WS_{158,3}$	$WS_{124,4}$	$WS_{133,1}$	$WS_{27,5}$	$WS_{337,5}$
8	$WS_{56,2}$	$WS_{53,7}$	$WS_{51,5}$	$WS_{15,10}$	$WS_{43,4}$	$WS_{147,3}$	$WS_{103,8}$	$WS_{194,7}$
9	$WS_{32,7}$	$WS_{36,2}$	$WS_{63,7}$	$WS_{63,3}$	$WS_{111,12}$	$WS_{165,5}$	$WS_{260,6}$	$WS_{398,7}$
10	$WS_{38,2}$	$WS_{41,3}$	$WS_{85,1}$	$WS_{135,8}$	$WS_{132,1}$	$WS_{201,6}$	$WS_{27,1}$	$WS_{243,3}$
11	$WS_{43,7}$	$WS_{31,1}$	$WS_{75,7}$	$WS_{38,4}$	$WS_{169,4}$	$WS_{72,2}$	$WS_{4,11}$	$WS_{145,3}$
12	$WS_{4,1}$	$WS_{61,7}$	$WS_{55,10}$	$WS_{149,9}$	$WS_{176,4}$	$WS_{93,7}$	$WS_{166,8}$	$WS_{21,5}$
13	$WS_{36,4}$	$WS_{41,5}$	$WS_{31,10}$	$WS_{16,1}$	$WS_{52,8}$	$WS_{81,5}$	$WS_{180,5}$	$WS_{44,6}$
14	$WS_{27,1}$	$WS_{68,8}$	$WS_{35,7}$	$WS_{165,8}$	$WS_{106,12}$	$WS_{197,6}$	$WS_{32,10}$	$WS_{298,8}$
15	$WS_{60,9}$	$WS_{60,5}$	$WS_{4,5}$	$WS_{179,9}$	$WS_{85,1}$	$WS_{235,2}$	$WS_{144,5}$	$WS_{385,8}$
16	$WS_{22,6}$	$WS_{16,8}$	$WS_{41,9}$	$WS_{133,5}$	$WS_{20,10}$	$WS_{244,2}$	$WS_{28,11}$	$WS_{6,5}$
17	$WS_{30,1}$	$WS_{90,1}$	$WS_{82,5}$	$WS_{93,6}$	$WS_{242,6}$	$WS_{228,2}$	$WS_{256,1}$	$WS_{92,3}$
18	$WS_{44,1}$	$WS_{33,1}$	$WS_{54,7}$	$WS_{90,11}$	$WS_{131,4}$	$WS_{248,5}$	$WS_{40,7}$	$WS_{97,7}$
19	$WS_{30,3}$	$WS_{66,3}$	$WS_{35,3}$	$WS_{127,12}$	$WS_{197,2}$	$WS_{269,1}$	$WS_{289,2}$	$WS_{42,7}$
20	$WS_{53,1}$	$WS_{77,6}$	$WS_{48,3}$	$WS_{45,4}$	$WS_{166,8}$	$WS_{35,4}$	$WS_{332,9}$	$WS_{119,4}$
21	$WS_{29,10}$	$WS_{25,3}$	$WS_{30,5}$	$WS_{79,7}$	$WS_{213,4}$	$WS_{31,4}$	$WS_{55,2}$	$WS_{195,6}$
22	$WS_{53,2}$	$WS_{83,5}$	$WS_{4,4}$	$WS_{16,6}$	$WS_{131,6}$	$WS_{199,4}$	$WS_{209,5}$	$WS_{300,2}$
23	$WS_{62,6}$	$WS_{64,2}$	$WS_{85,4}$	$WS_{124,10}$	$WS_{112,7}$	$WS_{134,2}$	$WS_{311,1}$	$WS_{167,5}$
24	$WS_{17,4}$	$WS_{17,3}$	$WS_{11,2}$	$WS_{1,1}$	$WS_{180,2}$	$WS_{75,8}$	$WS_{144,2}$	$WS_{140,3}$

**Table 6:** The accuracy of bearing fault classifications corresponding to each class.

Class	Epoch Length (Sample)							
	600	900	1200	3000	6000	9000	12,000	15,000
NORM	99.9996	100.0000	100.0000	100.0000	100.0000	100.0000	100.0000	100.0000
	±0.001	±0.0	±0.0	±0.0	±0.0	±0.0	±0.0	±0.0
BA07D	100.0000	100.0000	100.0000	100.0000	100.0000	100.0000	100.0000	100.0000
	±0.0	±0.0	±0.0	±0.0	±0.0	±0.0	±0.0	±0.0
BA14D	99.9914	100.0000	99.9997	100.0000	100.0000	100.0000	100.0000	100.0000
	±0.004	±0.0	±0.002	±0.0	±0.0	±0.0	±0.0	±0.0
BA21D	99.9931	100.0000	99.9997	100.0000	100.0000	100.0000	100.0000	100.0000
	±0.005	±0.0	±0.002	±0.0	±0.0	±0.0	±0.0	±0.0
BA28D	100.0000	100.0000	100.0000	100.0000	100.0000	100.0000	100.0000	100.0000
	±0.0	±0.0	±0.0	±0.0	±0.0	±0.0	±0.0	±0.0
BA07F	99.9726	99.9937	99.9965	100.0000	100.0000	100.0000	100.0000	100.0000
	±0.005	±0.0	±0.005	±0.0	±0.0	±0.0	±0.0	±0.0
BA14F	99.9911	99.9866	99.9963	99.9789	100.0000	100.0000	100.0000	100.0000
	±0.003	±0.002	±0.006	±0.000	±0.0	±0.0	±0.0	±0.0
BA21F	99.9954	99.9929	99.9991	99.9789	100.0000	100.0000	100.0000	100.0000
	±0.001	±0.002	±0.003	±0.000	±0.0	±0.0	±0.0	±0.0
IR07D	99.9997	100.0000	100.0000	100.0000	100.0000	100.0000	100.0000	100.0000
	±0.001	±0.0	±0.0	±0.0	±0.0	±0.0	±0.0	±0.0
IR14D	100.0000	100.0000	100.0000	100.0000	100.0000	100.0000	100.0000	100.0000
	±0.0	±0.0	±0.0	±0.0	±0.0	±0.0	±0.0	±0.0
IR21D	100.0000	100.0000	100.0000	100.0000	100.0000	100.0000	100.0000	100.0000
	±0.0	±0.0	±0.0	±0.0	±0.0	±0.0	±0.0	±0.0
IR28D	99.9961	100.0000	100.0000	100.0000	100.0000	100.0000	100.0000	100.0000
	±0.001	±0.0	±0.0	±0.0	±0.0	±0.0	±0.0	±0.0
IR07F	99.9933	100.0000	99.9994	100.0000	100.0000	100.0000	100.0000	100.0000
	±0.003	±0.0	±0.002	±0.0	±0.0	±0.0	±0.0	±0.0
IR14F	99.9994	100.0000	100.0000	100.0000	100.0000	100.0000	100.0000	100.0000
	±0.001	±0.0	±0.0	±0.0	±0.0	±0.0	±0.0	±0.0
IR21F	100.0000	100.0000	100.0000	100.0000	100.0000	100.0000	100.0000	100.0000
	±0.0	±0.0	±0.0	±0.0	±0.0	±0.0	±0.0	±0.0

**Table 7:** The precision of bearing fault classifications corresponding to each class.

Class	Epoch Length (Sample)							
	600	900	1200	3000	6000	9000	12,000	15,000
NORM	99.9927	100.0000	100.0000	100.0000	100.0000	100.0000	100.0000	100.0000
	±0.022	±0.0	±0.0	±0.0	±0.0	±0.0	±0.0	±0.0
BA07D	100.0000	100.0000	100.0000	100.0000	100.0000	100.0000	100.0000	100.0000
	±0.0	±0.0	±0.0	±0.0	±0.0	±0.0	±0.0	±0.0
BA14D	99.9108	100.0000	100.0000	100.0000	100.0000	100.0000	100.0000	100.0000
	±0.045	±0.0	±0.0	±0.0	±0.0	±0.0	±0.0	±0.0
BA21D	99.9235	100.0000	99.9957	100.0000	100.0000	100.0000	100.0000	100.0000
	±0.051	±0.0	±0.023	±0.0	±0.0	±0.0	±0.0	±0.0
BA28D	100.0000	100.0000	100.0000	100.0000	100.0000	100.0000	100.0000	100.0000
	±0.0	±0.0	±0.0	±0.0	±0.0	±0.0	±0.0	±0.0
BA07F	99.9742	100.0000	100.0000	100.0000	100.0000	100.0000	100.0000	100.0000
	±0.043	±0.0	±0.0	±0.0	±0.0	±0.0	±0.0	±0.0
BA14F	99.8680	99.8019	99.9444	99.6875	100.0000	100.0000	100.0000	100.0000
	±0.049	±0.032	±0.091	±0.0	±0.0	±0.0	±0.0	±0.0
BA21F	99.9936	100.0000	100.0000	100.0000	100.0000	100.0000	100.0000	100.0000
	±0.019	±0.0	±0.0	±0.0	±0.0	±0.0	±0.0	±0.0
IR07D	99.9958	100.0000	100.0000	100.0000	100.0000	100.0000	100.0000	100.0000
	±0.016	±0.0	±0.0	±0.0	±0.0	±0.0	±0.0	±0.0
IR14D	100.0000	100.0000	100.0000	100.0000	100.0000	100.0000	100.0000	100.0000
	±0.0	±0.0	±0.0	±0.0	±0.0	±0.0	±0.0	±0.0
IR21D	100.0000	100.0000	100.0000	100.0000	100.0000	100.0000	100.0000	100.0000
	±0.0	±0.0	±0.0	±0.0	±0.0	±0.0	±0.0	±0.0
IR28D	99.9422	100.0000	100.0000	100.0000	100.0000	100.0000	100.0000	100.0000
	±0.016	±0.0	±0.0	±0.0	±0.0	±0.0	±0.0	±0.0
IR07F	99.8997	100.0000	99.9914	100.0000	100.0000	100.0000	100.0000	100.0000
	±0.045	±0.0	±0.032	±0.0	±0.0	±0.0	±0.0	±0.0
IR14F	99.9914	100.0000	100.0000	100.0000	100.0000	100.0000	100.0000	100.0000
	±0.022	±0.0	±0.0	±0.0	±0.0	±0.0	±0.0	±0.0
IR21F	100.0000	100.0000	100.0000	100.0000	100.0000	100.0000	100.0000	100.0000
	±0.0	±0.0	±0.0	±0.0	±0.0	±0.0	±0.0	±0.0

**Table 8:** The recall of bearing fault classifications corresponding to each class.

Class	Epoch Length (Sample)							
	600	900	1200	3000	6000	9000	12,000	15,000
NORM	100.0000	100.0000	100.0000	100.0000	100.0000	100.0000	100.0000	100.0000
	±0.0	±0.0	±0.0	±0.0	±0.0	±0.0	±0.0	±0.0
BA07D	100.0000	100.0000	100.0000	100.0000	100.0000	100.0000	100.0000	100.0000
	±0.0	±0.0	±0.0	±0.0	±0.0	±0.0	±0.0	±0.0
BA14D	99.9617	100.0000	99.9957	100.0000	100.0000	100.0000	100.0000	100.0000
	±0.030	±0.0	±0.023	±0.0	±0.0	±0.0	±0.0	±0.0
BA21D	99.9745	100.0000	100.0000	100.0000	100.0000	100.0000	100.0000	100.0000
	±0.042	±0.0	±0.0	±0.0	±0.0	±0.0	±0.0	±0.0
BA28D	100.0000	100.0000	100.0000	100.0000	100.0000	100.0000	100.0000	100.0000
	±0.0	±0.0	±0.0	±0.0	±0.0	±0.0	±0.0	±0.0
BA07F	99.6164	99.9067	99.9483	100.0000	100.0000	100.0000	100.0000	100.0000
	±0.073	±0.000	±0.071	±0.0	±0.0	±0.0	±0.0	±0.0
BA14F	100.0000	100.0000	100.0000	100.0000	100.0000	100.0000	100.0000	100.0000
	±0.0	±0.0	±0.0	±0.0	±0.0	±0.0	±0.0	±0.0
BA21F	99.9379	99.8938	99.9871	99.6845	100.0000	100.0000	100.0000	100.0000
	±0.000	±0.033	±0.039	±0.0	±0.0	±0.0	±0.0	±0.0
IR07D	100.0000	100.0000	100.0000	100.0000	100.0000	100.0000	100.0000	100.0000
	±0.0	±0.0	±0.0	±0.0	±0.0	±0.0	±0.0	±0.0
IR14D	100.0000	100.0000	100.0000	100.0000	100.0000	100.0000	100.0000	100.0000
	±0.0	±0.0	±0.0	±0.0	±0.0	±0.0	±0.0	±0.0
IR21D	100.0000	100.0000	100.0000	100.0000	100.0000	100.0000	100.0000	100.0000
	±0.0	±0.0	±0.0	±0.0	±0.0	±0.0	±0.0	±0.0
IR28D	100.0000	100.0000	100.0000	100.0000	100.0000	100.0000	100.0000	100.0000
	±0.0	±0.0	±0.0	±0.0	±0.0	±0.0	±0.0	±0.0
IR07F	100.0000	100.0000	100.0000	100.0000	100.0000	100.0000	100.0000	100.0000
	±0.0	±0.0	±0.0	±0.0	±0.0	±0.0	±0.0	±0.0
IR14F	100.0000	100.0000	100.0000	100.0000	100.0000	100.0000	100.0000	100.0000
	±0.0	±0.0	±0.0	±0.0	±0.0	±0.0	±0.0	±0.0
IR21F	100.0000	100.0000	100.0000	100.0000	100.0000	100.0000	100.0000	100.0000
	±0.0	±0.0	±0.0	±0.0	±0.0	±0.0	±0.0	±0.0

**Table 9:** Comparison of methods and their performance of bearing fault classification using the CWRU bearing dataset.

Reference	Computational Method	Bearing Fault Condition	Performance
[41]	Processing: the multiscale large kernel feature extraction (MLKFE); Classification: few-shot learning model via an ensembling transformer-based model with Mahalanobis distance metric	Normal, 0.007-inch ball fault, 0.014-inch ball fault, 0.021-inch ball fault, 0.007-inch inner race fault, 0.014-inch inner race fault, 0.021-inch inner race fault, 0.007-inch outer race fault, 0.014-inch outer race fault, 0.021-inch outer race fault	Accurcy: 99.89 (best)
[6]	Processing: short-time Fourier transform; Classification: lite convolutional neural network (CNN) model with fixed feature map dimensions	Normal, 0.007-inch ball fault, 0.014-inch ball fault, 0.021-inch ball fault, 0.028-inch ball fault, 0.007-inch inner race fault, 0.014-inch inner race fault, 0.021-inch inner race fault, 0.028-inch inner race fault, 0.007-inch outer race fault, 0.014-inch outer race fault, 0.021-inch outer race fault	Accuracy: 99.93 (mean); 100.00 (max); 99.85 (min)
[13]	Processing: empirical mode decomposition (EMD) and cepstral autoregressive feature extraction; Classification: support vector machine	Normal, inner race fault, outer race fault	Accuracy: 98.70
[42]	Processing: feature extraction including maximum, minimum, mean, standard deviation, root mean square, skewness, kurtosis, crest factor, form factor; Classification: deep neural network with extreme gradient boosting optimization using particle swarm optimization (PSO)	Normal, 0.007-inch ball fault, 0.014-inch ball fault, 0.021-inch ball fault, 0.007-inch inner race fault, 0.014-inch inner race fault, 0.021-inch inner race fault, 0.007-inch outer race fault, 0.014-inch outer race fault, 0.021-inch outer race fault	Accuracy: 99.10
[43]	Classification: multi-scale convolutional neural network (CNN) and long short term memory (LSTM) model	Normal, 0.007-inch ball fault, 0.014-inch ball fault, 0.021-inch ball fault, 0.007-inch inner race fault, 0.014-inch inner race fault, 0.021-inch inner race fault, 0.007-inch 6 o’clock outer race fault, 0.014-inch 6 o’clock outer race fault, 0.021-inch 6 o’clock outer race fault	Accuracy: 98.46
[44]	Classification: Residual network with deformable convolution (DC-ResNet)	Normal, 0.007-inch ball fault, 0.014-inch ball fault, 0.021-inch ball fault, 0.007-inch inner race fault	Accuracy: 100.00 (achievable)
[45]	Classification: convolutional neural network (CNN) and recurrent neural network (RNN)	Normal, 0.007-inch ball fault, 0.014-inch ball fault, 0.021-inch ball fault, 0.007-inch inner race fault, 0.014-inch inner race fault, 0.021-inch inner race fault, 0.007-inch 6 o’clock outer race fault, 0.014-inch 6 o’clock outer race fault, 0.021-inch 6 o’clock outer race fault, 0.007-inch 3 o’clock outer race fault, 0.021-inch 3 o’clock outer race fault, 0.007-inch 12 o’clock outer race fault, 0.021-inch 12 o’clock outer race fault	Accuracy: 99.32 (0-HP motor load); 91.87 (1-HP motor load); 94.97 (2-HP motor load)
[46]	Classification: hybrid models based on convolutional neural network (CNN) and various classifiers, including random forest, gcForest, support vector machine, long short term memory	Normal, 0.007-inch ball fault, 0.014-inch ball fault, 0.021-inch ball fault, 0.007-inch inner race fault, 0.014-inch inner race fault, 0.021-inch inner race fault, 0.007-inch outer race fault, 0.014-inch outer race fault, 0.021-inch outer race fault	Accuracy: 98.90 (CNN-RF); 99.10 (CNN-gcForest); 99.00 (CNN-SVM); 98.67 (CNN-LSTM)
[47]	Classification: Siamese neural network-based deep convolutional neural networks with wide first-layer kernel (WDCNN)	Normal, 0.007-inch ball fault, 0.014-inch ball fault, 0.021-inch ball fault, 0.007-inch inner race fault, 0.014-inch inner race fault, 0.021-inch inner race fault, 0.007-inch outer race fault, 0.014-inch outer race fault, 0.021-inch outer race fault	Accuracy: 99.65 (WDCNN); 99.77 (five-shot); 99.79 (one-shot)
This study	Processing: wavelet scattering transform Classification: support vector machine (SVM) with the second-order polynomial kernel function	Normal; Drive end: 0.007-inch, 0.014-inch, 0.021-inch, 0.028-inch ball faults, 0.007-inch, 0.014-inch, 0.021-inch, 0.028-inch inner race faults; Fan end: 0.007-inch, 0.014-inch, 0.021-inch ball faults, 0.007-inch, 0.014-inch, 0.021-inch inner race faults	Accuracy: 100.00

## Data Availability

The data used in this study are publicly available online at Bearing Data Center, Case Western Reserve University https://engineering.case.edu/bearingdatacenter.

## References

[1] Janjarasjitt S., Ocak H., Loparo K.A. (2008). Bearing condition diagnosis and prognosis using applied nonlinear dynamical analysis of machine vibration signal. J. Sound Vib..

[2] Kannan V., Zhang T., Li H. (2024). A review of the intelligent condition monitoring of rolling element bearings. Machines.

[3] Hamadache M., Hung J.H., Park J., You B.D. (2019). A comprehensive review of artificial intelligence-based approaches for rolling element bearing PHM: Shallow and deep learning. JMST Adv..

[4] Tandon T., Choudhury A. (1999). A review of vibration and acoustic measurement methods for the detection of defects in rolling element bearings. Tribol. Int..

[5] McFadden P.D., Smith J.D. (1984). Vibration monitoring of rolling element bearings by high frequency resonance technique—A review. Tribol. Int..

[6] Yoo Y., Jo H., Ban S.W. (2023). Lite and efficient deep learning model for bearing fault diagnosis using the CWRU dataset. Sensors.

[7] Lee J.H., Kim J., Kim H.J. (2001). Development of enhanced Wigner-Ville distribution function. Mech. Syst. Signal Process..

[8] Peng Z., Chu F., He Y. (2002). Vibration signal analysis and feature extraction based on reassigned wavelet scalogram. J. Sound Vib..

[9] Qiu H., Lee J., Linb J., Yu G. (2006). Wavelet filter-based weak signature detection method and its application on rolling element bearing prognostics. J. Sound Vib..

[10] Seker S., Ayaz E. (2003). Feature extraction related to bearing damage in electric motors by wavelet analysis. J. Frankl. Inst..

[11] Li C.J., Ma J. (1997). Wavelet decomposition of vibrations for detection of bearing localized defects. NDT E Int..

[12] Castejon C., Gomez M.J., Garcia-Prada J.C., Ordonez A.J., Rubio H. (2015). Automatic selection of the WPT decomposition level for condition monitoring of rotor elements based on the sensitivity analysis of the wavelet energy. Int. J. Acoust. Vib..

[13] Aziz S., Khan M.U., Faraz M., Montes G.A. (2023). Intelligent bearing faults diagnosis featuring automated relative energy based empirical mode decomposition and novel cepstral autoregressive features. Measurement.

[14] Neupane D., Seok J. (2020). Bearing fault detection and diagnosis using Case Western Reserve University dataset with deep learning approaches: A review. IEEE Access.

[15] Ocak H., Loparo K.A. (2005). An HMM based fault detection and diagnosis scheme for rolling element bearings. J. Vib. Acoust..

[16] Ocak H., Loparo K.A., Discenzo F.M. (2007). Online tracking of bearing wear using wavelet packet decomposition and probabilistic modeling: A method for bearing prognostics. J. Sound Vib..

[17] Yang J., Zhang Y., Zhu Y. (2007). Intelligent fault diagnosis of rolling element bearing based on SVMs and fractal dimension. Mech. Syst. Signal Process..

[18] Wang Z., Yao L., Cai Y. (2020). Rolling bearing fault diagnosis using generalized refined composite multiscale sample entropy and optimized support vector machine. Measurement.

[19] Hwang D., Youn Y., Sun J., Choi K., Lee J., Kim Y. (2015). Support vector machine based bearing fault diagnosis for induction motors using vibration signals. J. Electr. Eng. Technol..

[20] Li Y., Xu M., Wei Y., Huang W. (2016). A new rolling bearing fault diagnosis method based on multiscale permutation entropy and improved support vector machine based binary tree. Measurement.

[21] Seera M., Wong M.L.D., Nandi A.K. (2017). Classification of ball bearing faults using a hybrid intelligent model. Appl. Soft Comput..

[22] Vakharia V., Gupta V.K., Kankar P.K. (2017). Efficient fault diagnosis of ball bearing using reliefF and random forest classifier. J. Braz. Soc. Mech. Sci. Eng..

[23] Pandya D.H., Upadhyay S.H., Harsha S. (2013). Fault diagnosis of rolling element bearing with intrinsic mode function of acoustic emission data using APF-KNN. Expert Syst. Appl..

[24] Kumar H.S., Upadhyaya G. (2023). Fault diagnosis of rolling element bearing using continuous wavelet transform and K-nearest neighbour. Mater. Today Proc..

[25] Wang Q., Liu Y., He X., Liu S., Liu J. (2014). Fault diagnosis of bearing based on KPCA and KNN method. Adv. Mater. Res..

[26] Eren L., Ince T., Kiranyaz S. (2019). A generic intelligent bearing fault diagnosis system using compact adaptive 1D CNN classifier. J. Signal Process. Syst..

[27] Zhang H., Shi P., Han D., Jia L. (2023). Research on rolling bearing fault diagnosis method based on AMVMD and convolutional neural networks. Measurement.

[28] Gao D., Zhu Y., Ren Z., Yan K., Kang W. (2021). A novel weak fault diagnosis method for rolling bearings based on LSTM considering quasi-periodicity. Knowl.-Based Syst..

[29] Liang M., Zhou K. (2023). Joint loss learning-enabled semi-supervised autoencoder for bearing fault diagnosis under limited labeled vibration signals. J. Vib. Control..

[30] Mallat S. (2016). Understanding deep convolutional networks. Philos. Trans. R. Soc. A Math. Phys. Eng. Sci..

[31] Anden J., Mallat S. (2014). Deep scattering spectrum. IEEE Trans. Signal Process..

[32] Bruna J., Mallat S. (2013). Invariant scattering convolution networks. IEEE Trans. Pattern Anal. Mach. Intell..

[33] Mallat S. (2012). Group invariant scattering. Commun. Pure Appl. Math..

[34] LeCun Y., Boser B., Denker J.S., Henderson D., Howard R.R., Hubbard W., Jackel L.D., Touretzky D. (1990). Handwritten Digit Recognition with a Back-Propagation Network. Advances in Neural Information Processing Systems (NIPS 1989).

[35] Ding C., Peng H. (2005). Minimum redundancy feature selection from microarray gene expression data. J. Bioinform. Comput. Biol..

[36] Boser B.E., Guyon I.M., Vapnik V.N. A training algorithm for optimal margin classifiers. Proceedings of the Fifth Annual Workshop on Computational Learning Theory, COLT ’92.

[37] Cortes C., Vapnik V. (1995). Support-vector networks. Mach. Learn..

[38] Vapnik V.N. (2013). The Nature of Statistical Learning Theory.

[39] Cristianini N., Ricci E., Kao M.Y. (2008). Support Vector Machines. Encyclopedia of Algorithms.

[40] Schölkopf B., Smola A.J. (2001). Support Vector Machines.

[41] Vu M.H., Nguyen V.Q., Tran T.T., Pham V.T., Lo M.T. (2024). Few-shot bearing fault diagnosis via ensembling transformer-based model with Mahalanobis distance metric learning from multiscale features. IEEE Trans. Instrum. Meas..

[42] Lee C.Y., Maceren E.D.C. (2024). Induction motor bearing fault classification using deep neural network with particle swarm optimization-extreme gradient boosting. IET Electr. Power Appl..

[43] Chen X., Zhang B., Gao D. (2021). Bearing fault diagnosis based on multi-scale CNN and LSTM model. J. Intell. Manuf..

[44] Zhao Y., Zhou M., Xu X., Zhang N. (2023). Fault diagnosis of rolling bearings with noise signal based on modified kernel principal component analysis and DC-ResNet. CAAI Trans. Intell. Technol..

[45] Raj K.K., Kumar S., Kumar R.R., Andriollo M. (2024). Enhanced fault detection in bearings using machine learning and raw accelerometer data: A case study using the Case Western Reserve University dataset. Information.

[46] Xie W., Li Z., Xu Y., Gardoni P., Li W. (2022). Evaluation of different bearing fault classifiers in utilizing CNN feature extraction ability. Sensors.

[47] Zhang A., Li S., Cui Y., Yang W., Dong R., Hu J. (2019). Limited data rolling bearing fault diagnosis with few-shot learning. IEEE Access.

